# Supplementary material for: Parent Involvement in Diet or Physical Activity Interventions to Treat or Prevent Childhood Obesity: An Umbrella Review
Source: Nutrients. 2021 Sep 16;13(9):3227. doi: 10.3390/nu13093227 (PMC8464903; doi:10.3390/nu13093227)
Supplement: Supplementary file 1 [file nutrients-13-03227-s001.zip › nutrients-1320147-supplementary/Supplementary Files/Table S1. Search Strategy.pdf]

Supplementary Table S1: Search Strategy

All searches were conducted in January 2020. Searches were customized for each database to include appropriate keywords and controlled vocabulary terms related to the topic of parental or caregiver involvement in interventions to control or treat childhood obesity. Filters designed to identify systematic reviews and meta-analyses were applied, as were English language filters.

Ovid/MEDLINE

69	limit 68 to (english and (meta analysis or systematic reviews))
68	10 and 23 and 53 and 67
67	or/54-66
66	guardian*.ti,ab,kw.
65	(care adj2 (giver* or provider*)).ti,ab,kw.
64	carer*.ti,ab,kw.
63	caregiver*.ti,ab,kw.
62	"family based".ti,ab,kw.
61	father*.ti,ab,kw.
60	mother*.ti,ab,kw.
59	paternal.ti,ab,kw.
58	maternal.ti,ab,kw.
57	parent*.ti,ab,kw.
56	exp Caregivers/
55	exp PARENTING/
54	exp PARENTS/
53	32 or 52
52	or/33-51
51	(Behavio?r adj2 chang*).ab,kw,ti.
50	(physical adj2 (activit* or fitness or training or conditioning or exertion)).ab,kw,ti.
49	"calori*".ab,kwzz
48	"sedentar*".ab,kw,ti.
47	"exercis*".ab,kw,ti.
46	"snack*".ab,kw,ti.
45	appetite.ab,kw,ti.
44	"meal*".ab,kw,ti.
43	"feeding*".ab,kw,ti.
42	"food*".ab,kw,ti.
41	intake.ab,kw,ti.
40	eating.ab,kw,ti.
39	nutritive.ab,kw,ti.
38	nutrition.ab,kw,ti.
37	"diet*".ab,kw,ti.
36	"program*".ab,kw,ti.
35	"intervention*".ab,kw,ti.
34	reduc*.ti,ab,kw.
33	prevent*.ti,ab,kw.

32	or/24-31
31	exp Life Style/
30	exp Physical Fitness/
29	exp EXERCISE/
28	exp FOOD/ or exp FOOD PREFERENCES/
27	exp "diet, food, and nutrition"/
26	exp Risk Reduction Behavior/
25	exp Preventive Health Services/
24	prevention & control.fs.
23	or/11-22
22	("out of school time" or "after school").ti,ab,kw.
21	(childcare or "child care").ti,ab,kw.
20	preschool*.ti,ab,kw.
19	((elementary or primary or grade or grammar or pre) adj2 school*).ti,ab,kw.
18	(juvenile* or youth*).ti,ab,kw.
17	p?ediatric*.ti,ab,kw.
16	(child* or girl* or boy*).ti,ab,kw.
15	(adolescen* or pubert* or pubescen* or teen*).ti,ab,kw.
14	(preadolescen* or prepubert* or prepubescen* or preteen*).ti,ab,kw.
13	(pre adj1 (adolescen* or pubert* or pubescen* or teen* or school*)).ti,ab,kw.
12	Pediatrics/
11	exp CHILD/
10	1 or 2 or 3 or 4 or 5 or 6 or 7 or 8 or 9
9	(weight adj2 (reduc* or loss or lose or lost or losing or gain* or increas* or chang* or related)).ti,ab,kw.
8	(bmi or "body mass").ti,ab,kw.
7	(overnutrition or "over nutrition").ti,ab,kw.
6	adipos*.ti,ab,kw.
5	overweight.ti,ab,kw.
4	obes*.ti,ab,kw.
3	exp Child Nutrition Disorders/
2	exp Body Mass Index/
1	exp OVERNUTRITION/

Cochrane Library

D	Search
#1	Any MeSH descriptor with qualifier(s): [Prevention & control - PC]
#2	[mh "Preventive Health Services"]
#3	[mh "Risk Reduction Behavior"]
#4	[mh "Diet, Food, and Nutrition"]
#5	[mh Food]
#6	[mh "Food Preferences"]
#7	[mh Exercise]
#8	[mh "Physical Fitness"]
#9	[mh "Life Style"]
#10	{or #1-#9}
#11	prevent*:ti,ab,kw
#12	reduc*:ti,ab,kw

#13	intervention*:ti,ab,kw
#14	program*:ti,ab,kw
#15	diet*:ti,ab,kw
#16	nutrition*:ti,ab,kw
#17	nutritive:ti,ab,kw
#18	eating:ti,ab,kw
#19	intake:ti,ab,kw
#20	food*:ti,ab,kw
#21	feeding:ti,ab,kw
#22	meal*:ti,ab,kw
#23	appetite*:ti,ab,kw
#24	snack*:ti,ab,kw
#25	exercis*:ti,ab,kw
#26	sedentar*:ti,ab,kw
#27	calori*:ti,ab,kw
#28	physical near/2 (activit* or fitness or training or conditioning or exertion):ti,ab,kw
#29	Behavio?r near/2 chang*:ti,ab,kw
#30	{or #11-#29}
#31	#10 or #30
#32	pre next (adolescen* or pubert* or pubescen* or teen* or school*):ti,ab,kw
#33	(adolescen* or pubert* or pubescen* or teen*):ti,ab,kw
#34	(preadolescen* or prepubert* or prepubescen* or preteen*):ti,ab,kw
#35	child*:ti,ab,kw
#36	girl*:ti,ab,kw
#37	boy*:ti,ab,kw
#38	(paediatric* or pediatric*):ti,ab,kw
#39	youth*:ti,ab,kw
#40	juvenile*:ti,ab,kw
#41	((elementary or primary or grade or grammar or pre) near/2 school*):ti,ab,kw
#42	preschool:ti,ab,kw
#43	"after school":ti,ab,kw
#44	"out of school time":ti,ab,kw
#45	childcare:ti,ab,kw
#46	"child care":ti,ab,kw
#47	MeSH descriptor: [Pediatrics] explode all trees
#48	MeSH descriptor: [Child] explode all trees
#49	{or #32-#48}
#50	MeSH descriptor: [Overnutrition] explode all trees
#51	MeSH descriptor: [Child Nutrition Disorders] explode all trees
#52	MeSH descriptor: [Body Mass Index] explode all trees
#53	obes*:ti,ab,kw
#54	overweight:ti,ab,kw
#55	"over weight":ti,ab,kw
#56	(weight near/2 (reduc* or loss or lose or lost or losing or gain* or increas* or chang* or related)):ti,ab,kw
#57	adipos*:ti,ab,kw
#58	overnutrition:ti,ab,kw
#59	"over nutrition":ti,ab,kw
#60	BMI*:ti,ab,kw

#61	"body mass":ti,ab,kw
#62	{or #50-#61}
#63	MeSH descriptor: [Parents] explode all trees
#64	MeSH descriptor: [Parenting] explode all trees
#65	MeSH descriptor: [Caregivers] explode all trees
#66	parent*:ti,ab,kw
#67	maternal:ti,ab,kw
#68	paternal:ti,ab,kw
#69	mother*:ti,ab,kw
#70	father*ti,ab,kw
#71	"family based":ti,ab,kw
#72	caregiver*:ti,ab,kw
#73	carer*:ti,ab,kw
#74	(care near/2 (giver* or provider*)):ti,ab,kw
#75	guardian*:ti,ab,kw
#76	{or #63-#75}
#77	#31 and #49 and #62 and #76
#78	#31 and #49 and #62 and #76 in Other Reviews

Embase

No.	Query
#75	#15 AND #28 AND #59 AND #72 AND [english]/lim AND ([cochrane review]/lim OR [sy review]/lim OR [meta analysis]/lim)
#74	#15 AND #28 AND #59 AND #72 AND [english]/lim
#73	#15 AND #28 AND #59 AND #72
#72	#60 OR #61 OR #62 OR #63 OR #64 OR #65 OR #66 OR #67 OR #68 OR #69 OR #70 OR #71
#71	guardian*:ti,ab
#70	(care NEAR/2 (giver* OR provider*)):ti,ab
#69	carer*:ti,ab
#68	caregiver*:ti,ab
#67	'family based':ti,ab
#66	father*:ti,ab
#65	mother*:ti,ab

No.	Query
#64	paternal:ti,ab
#63	maternal:ti,ab
#62	parent*:ti,ab,kw
#61	'caregiver'/exp
#60	'parent'/exp
#59	#38 OR #58
#58	#39 OR #40 OR #41 OR #42 OR #43 OR #44 OR #45 OR #46 OR #47 OR #48 OR #49 OR #50 OR #51 OR #52 OR #53 OR #54 OR #55 OR #56 OR #57
#57	((behavior OR behaviour) NEAR/2 chang*):ti,ab
#56	(physical NEAR/2 (activit* OR fitness OR training OR conditioning OR exertion)):ti,ab
#55	calori*:ti,ab
#54	sedentar*:ti,ab
#53	exercis*:ti,ab
#52	snack*:ti,ab
#51	appetite*:ti,ab
#50	meal*:ti,ab
#49	feeding*:ti,ab
#48	food*:ti,ab
#47	intake:ti,ab
#46	eating:ti,ab
#45	nutritive:ti,ab
#44	nutrition*:ti,ab

No.	Query
#43	diet*:ti,ab
#42	intervention*:ti,ab
#41	program*:ti,ab
#40	reduc*:ti,ab
#39	prevent*:ti,ab
#38	#29 OR #30 OR #31 OR #32 OR #33 OR #34 OR #35 OR #36 OR #37
#37	'behavior change'/exp
#36	'lifestyle modification'/exp
#35	'physical activity, capacity and performance'/exp
#34	'food'/exp
#33	'feeding'/exp
#32	'nutrition'/exp
#31	'body weight management'/exp
#30	'risk reduction'/exp
#29	'prevention'/exp
#28	#16 OR #17 OR #18 OR #19 OR #20 OR #21 OR #22 OR #23 OR #24 OR #25 OR #26 OR #27
#27	childcare:ti,ab,kw OR 'child care':ti,ab,kw OR 'out of school time':ti,ab,kw OR 'after school':ti,ab,kw
#26	((elementary OR primary OR grade OR grammar OR pre) NEAR/2 school*):ti,ab,kw
#25	youth*:ti,ab,kw OR juvenile*:ti,ab,kw
#24	paediatric*:ti,ab,kw OR pediatric*:ti,ab,kw
#23	child*:ti,ab,kw OR girl*:ti,ab,kw OR boy*:ti,ab,kw

No.	Query
#22	preadolescen*:ti,ab,kw OR prepubert*:ti,ab,kw OR prepubescen*:ti,ab,kw OR preteen*:ti,ab,kw OR prerschool*:ti,ab,kw
#21	('pre' NEXT/1 (adolescen* OR pubert* OR pubescen* OR teen* OR school*)):ti,ab,kw
#20	adolescen*:ti,ab,kw OR pubert*:ti,ab,kw OR pubescen*:ti,ab,kw OR teen*:ti,ab,kw
#19	'pediatrics'/exp
#18	'childhood'/exp
#17	'child'/exp
#16	'child care'/exp
#15	#5 OR #14
#14	#6 OR #7 OR #8 OR #9 OR #10 OR #11 OR #12 OR #13
#13	overweight:ti,ab,kw OR 'over weight':ti,ab,kw
#12	(weight NEAR/2 (reduc* OR loss OR lose OR lost OR losing OR gain* OR increas* OR chang* OR related)):ti,ab,kw
#11	'body mass':ti,ab,kw
#10	bmi*:ti,ab,kw
#9	'over nutrition':ti,ab,kw
#8	overnutrition:ti,ab,kw
#7	adipos*:ti,ab,kw
#6	obes*:ti,ab,kw
#5	#1 OR #2 OR #3 OR #4
#4	'body weight management'/exp
#3	'body weight loss'/exp
#2	'overnutrition'/exp

No.	Query
#1	'body mass'/exp

Web of Science

# 34	#31 AND #28<br>**Refined by: LANGUAGES:** (ENGLISH) AND [excluding] **DOCUMENT TYPES:** (BOOK CHAPTER OR MEETING ABSTRACT OR EDITORIAL MATERIAL)<br>*Indexes=SCI-EXPANDED, SSCI, A&HCI, CPCI-S, CPCI-SSH, BKCI-S, BKCI-SSH, CCR-EXPANDED, IC Timespan=1900-2018*
# 33	#31 AND #28<br>**Refined by: LANGUAGES:** (ENGLISH)<br>*Indexes=SCI-EXPANDED, SSCI, A&HCI, CPCI-S, CPCI-SSH, BKCI-S, BKCI-SSH, CCR-EXPANDED, IC Timespan=1900-2018*
# 32	#31 AND #28<br>*Indexes=SCI-EXPANDED, SSCI, A&HCI, CPCI-S, CPCI-SSH, BKCI-S, BKCI-SSH, CCR-EXPANDED, IC Timespan=1900-2018*
# 31	#30 OR #29<br>*Indexes=SCI-EXPANDED, SSCI, A&HCI, CPCI-S, CPCI-SSH, BKCI-S, BKCI-SSH, CCR-EXPANDED, IC Timespan=1900-2018*
# 30	TS=(systematic NEAR/3 review*)<br>*Indexes=SCI-EXPANDED, SSCI, A&HCI, CPCI-S, CPCI-SSH, BKCI-S, BKCI-SSH, CCR-EXPANDED, IC Timespan=1900-2018*
# 29	TS=(metaanalys* OR meta-analys* OR "meta analys*")<br>*Indexes=SCI-EXPANDED, SSCI, A&HCI, CPCI-S, CPCI-SSH, BKCI-S, BKCI-SSH, CCR-EXPANDED, IC Timespan=1900-2018*
# 28	#27 AND #26 AND #25 AND #24<br>*Indexes=SCI-EXPANDED, SSCI, A&HCI, CPCI-S, CPCI-SSH, BKCI-S, BKCI-SSH, CCR-EXPANDED, IC Timespan=1900-2018*
# 27	#23 OR #22 OR #21 OR #20

	Indexes=SCI-EXPANDED, SSCI, A&HCI, CPCI-S, CPCI-SSH, BKCI-S, BKCI-SSH, CCR-EXPANDED, IC Timespan=1900-2018
# 26	#19 OR #18 OR #17 OR #16 OR #15<br>*Indexes=SCI-EXPANDED, SSCI, A&HCI, CPCI-S, CPCI-SSH, BKCI-S, BKCI-SSH, CCR-EXPANDED, IC Timespan=1900-2018*
# 25	#14 OR #13 OR #12 OR #11 OR #10 OR #9 OR #8 OR #7<br>*Indexes=SCI-EXPANDED, SSCI, A&HCI, CPCI-S, CPCI-SSH, BKCI-S, BKCI-SSH, CCR-EXPANDED, IC Timespan=1900-2018*
# 24	#6 OR #5 OR #4 OR #3 OR #2 OR #1<br>*Indexes=SCI-EXPANDED, SSCI, A&HCI, CPCI-S, CPCI-SSH, BKCI-S, BKCI-SSH, CCR-EXPANDED, IC Timespan=1900-2018*
# 23	TS=((behavior OR behaviour) NEAR/2 chang*)<br>*Indexes=SCI-EXPANDED, SSCI, A&HCI, CPCI-S, CPCI-SSH, BKCI-S, BKCI-SSH, CCR-EXPANDED, IC Timespan=1900-2018*
# 22	TS=(physical NEAR/2 (activit* or fitness or training or conditioning or exertion))<br>*Indexes=SCI-EXPANDED, SSCI, A&HCI, CPCI-S, CPCI-SSH, BKCI-S, BKCI-SSH, CCR-EXPANDED, IC Timespan=1900-2018*
# 21	TS=(eating OR intake OR food* OR feeding OR meal* OR appetite OR snack* OR exercise* OR sedentar* OR calori*)<br>*Indexes=SCI-EXPANDED, SSCI, A&HCI, CPCI-S, CPCI-SSH, BKCI-S, BKCI-SSH, CCR-EXPANDED, IC Timespan=1900-2018*
# 20	TS=(prevent* OR reduc* OR intervention* OR program* OR diet* OR nutrition* OR nutritive)<br>*Indexes=SCI-EXPANDED, SSCI, A&HCI, CPCI-S, CPCI-SSH, BKCI-S, BKCI-SSH, CCR-EXPANDED, IC Timespan=1900-2018*
# 19	TS=(care NEAR/2 (giver* OR provider*))<br>*Indexes=SCI-EXPANDED, SSCI, A&HCI, CPCI-S, CPCI-SSH, BKCI-S, BKCI-SSH, CCR-EXPANDED, IC Timespan=1900-2018*
# 18	TS=(caregiver* OR carer*)<br>*Indexes=SCI-EXPANDED, SSCI, A&HCI, CPCI-S, CPCI-SSH, BKCI-S, BKCI-SSH, CCR-EXPANDED, IC Timespan=1900-2018*
# 17	TS="family based"

	Indexes=SCI-EXPANDED, SSCI, A&HCI, CPCI-S, CPCI-SSH, BKCI-S, BKCI-SSH, CCR-EXPANDED, IC Timespan=1900-2018
# 16	TS=(maternal OR paternal)<br>*Indexes=SCI-EXPANDED, SSCI, A&HCI, CPCI-S, CPCI-SSH, BKCI-S, BKCI-SSH, CCR-EXPANDED, IC Timespan=1900-2018*
# 15	TS=(parent* OR mother* OR father* OR guardian*)<br>*Indexes=SCI-EXPANDED, SSCI, A&HCI, CPCI-S, CPCI-SSH, BKCI-S, BKCI-SSH, CCR-EXPANDED, IC Timespan=1900-2018*
# 14	TS=(childcare or "child care")<br>*Indexes=SCI-EXPANDED, SSCI, A&HCI, CPCI-S, CPCI-SSH, BKCI-S, BKCI-SSH, CCR-EXPANDED, IC Timespan=1900-2018*
# 13	TS=("after school" OR "out of school time")<br>*Indexes=SCI-EXPANDED, SSCI, A&HCI, CPCI-S, CPCI-SSH, BKCI-S, BKCI-SSH, CCR-EXPANDED, IC Timespan=1900-2018*
# 12	TS=((elementary OR primary OR grade OR grammar OR pre) NEAR/2 (school*))<br>*Indexes=SCI-EXPANDED, SSCI, A&HCI, CPCI-S, CPCI-SSH, BKCI-S, BKCI-SSH, CCR-EXPANDED, IC Timespan=1900-2018*
# 11	TS=(youth* OR juvenile*)<br>*Indexes=SCI-EXPANDED, SSCI, A&HCI, CPCI-S, CPCI-SSH, BKCI-S, BKCI-SSH, CCR-EXPANDED, IC Timespan=1900-2018*
# 10	TS=(pediatric* OR paediatric*)<br>*Indexes=SCI-EXPANDED, SSCI, A&HCI, CPCI-S, CPCI-SSH, BKCI-S, BKCI-SSH, CCR-EXPANDED, IC Timespan=1900-2018*
# 9	TS=(child* OR boy* OR girl*)<br>*Indexes=SCI-EXPANDED, SSCI, A&HCI, CPCI-S, CPCI-SSH, BKCI-S, BKCI-SSH, CCR-EXPANDED, IC Timespan=1900-2018*
# 8	TS=((pre) NEAR/1 (adolescen* OR pubert* OR pubescen* OR teen* OR school*))<br>*Indexes=SCI-EXPANDED, SSCI, A&HCI, CPCI-S, CPCI-SSH, BKCI-S, BKCI-SSH, CCR-EXPANDED, IC Timespan=1900-2018*
# 7	TS=(preadolescen* OR prepubert* OR prepubescen* OR preteen* OR preschool* OR adolescen* OR pubert* OR pubescen* OR teen*)

	Indexes=SCI-EXPANDED, SSCI, A&HCI, CPCI-S, CPCI-SSH, BKCI-S, BKCI-SSH, CCR-EXPANDED, IC Timespan=1900-2018
# 6	TS=("body mass" OR BMI)<br>*Indexes=SCI-EXPANDED, SSCI, A&HCI, CPCI-S, CPCI-SSH, BKCI-S, BKCI-SSH, CCR-EXPANDED, IC Timespan=1900-2018*
# 5	TS=(overnutrition OR "over nutrition")<br>*Indexes=SCI-EXPANDED, SSCI, A&HCI, CPCI-S, CPCI-SSH, BKCI-S, BKCI-SSH, CCR-EXPANDED, IC Timespan=1900-2018*
# 4	TS= ((weight) NEAR/2 (reduc* OR loss OR lose OR lost OR losing OR gain* OR increas* OR chang* OR related))<br>*Indexes=SCI-EXPANDED, SSCI, A&HCI, CPCI-S, CPCI-SSH, BKCI-S, BKCI-SSH, CCR-EXPANDED, IC Timespan=1900-2018*
# 3	TS=adipos*<br>*Indexes=SCI-EXPANDED, SSCI, A&HCI, CPCI-S, CPCI-SSH, BKCI-S, BKCI-SSH, CCR-EXPANDED, IC Timespan=1900-2018*
# 2	TS=(overweight OR "over weight")<br>*Indexes=SCI-EXPANDED, SSCI, A&HCI, CPCI-S, CPCI-SSH, BKCI-S, BKCI-SSH, CCR-EXPANDED, IC Timespan=1900-2018*
# 1	TS=obes*<br>*Indexes=SCI-EXPANDED, SSCI, A&HCI, CPCI-S, CPCI-SSH, BKCI-S, BKCI-SSH, CCR-EXPANDED, IC Timespan=1900-2018*

CINAHL

#	Query
S69	S28 AND S43 AND S54 AND S67 Limiters - Publication Type: Meta Analysis, Meta Synthesis, Systematic Review; Language: English
S68	S28 AND S43 AND S54 AND S67
S67	S55 OR S56 OR S57 OR S58 OR S59 OR S60 OR S61 OR S62 OR S63 OR S64 OR S65 OR S66
S66	guardian*
S65	care N2 (giver* OR provider*)
S64	carer*
S63	caregiver*
S62	"family based"
S61	father*
S60	mother*
S59	paternal

S58	maternal
S57	parent*
S56	(MH "Caregivers")
S55	(MH "Parents+")
S54	S44 OR S45 OR S46 OR S47 OR S48 OR S49 OR S50 OR S51 OR S52 OR S53
S53	"body mass"
S52	bmi*
S51	"over nutrition"
S50	overnutrition
S49	adipos*
S48	(weight N2 (reduc* OR loss OR lose OR lost OR losing OR gain* OR increas* OR chang* OR related)
S47	overweight
S46	obes*
S45	(MH "Obesity+")
S44	(MH "Body Mass Index")
S43	S29 OR S30 OR S31 OR S32 OR S33 OR S34 OR S35 OR S36 OR S37 OR S38 OR S39 OR S40 OR S41 OR S42
S42	(MH "Pediatric Care+")
S41	(MH "Child+")
S40	(MH "Pediatrics+")
S39	"out of school time"
S38	("after school") OR childcare OR "child care"
S37	(elementary OR primary OR grade OR grammar OR pre) N2 school*
S36	youth* OR juvenile*
S35	preschool*
S34	pediatric* OR paediatric*
S33	boy*
S32	girl*
S31	child*
S30	(adolescen* OR pubert* OR pubescen* OR teen*) OR (preadolescen* OR prepubert* OR prepubescen* OR preteen*)
S29	'pre' W1(adolescen* OR pubert* OR pubescen* OR teen* OR school*)
S28	S7 OR S27
S27	S8 OR S9 OR S10 OR S11 OR S12 OR S13 OR S14 OR S15 OR S16 OR S17 OR S18 OR S19 OR S20 OR S21 OR S22 OR S23 OR S24 OR S25 OR S26
S26	(behavior* OR behaviour*) N2 chang*
S25	physical N2 (activit* or fitness or training or conditioning or exertion)
S24	calori*
S23	sedentar*
S22	exercis*
S21	snack*
S20	appetite*
S19	meal*
S18	feeding
S17	food*
S16	intake
S15	eating
S14	nutritive

S13	nutrition*
S12	diet*
S11	program*
S10	intervention*
S9	reduc*
S8	prevent*
S7	(S1 OR S2 OR S3 OR S4 OR S5 OR S6)
S6	(MH "Life Style+")
S5	(MH "Exercise+") OR (MH "Physical Fitness+")
S4	(MH "Food Preferences")
S3	(MH "Food+")
S2	(MH "Diet+")
S1	(MH "Preventive Health Care+")

PsycInfo

#	Query
S60	S21 AND S34 AND S45 AND S58 Limiters - Language: English; Methodology: - Systematic Review, META ANALYSIS, METASYNTHESIS
S59	S21 AND S34 AND S45 AND S58
S58	S46 OR S47 OR S48 OR S49 OR S50 OR S51 OR S52 OR S53 OR S54 OR S55 OR S56 OR S57
S57	guardian*
S56	care N2 (giver* OR provider*)
S55	carer*
S54	caregiver*
S53	"family based"
S52	father*
S51	mother*
S50	paternal
S49	maternal
S48	parent*
S47	DE "Caregivers"
S46	(DE "Parents" OR DE "Adoptive Parents" OR DE "Fathers" OR DE "Foster Parents" OR DE "Mothers" OR DE "Single Parents" OR DE "Stepparents")
S45	S35 OR S36 OR S37 OR S38 OR S39 OR S40 OR S41 OR S42 OR S43 OR S44
S44	"body mass"
S43	bmi*
S42	"over nutrition"
S41	overnutrition
S40	adipos*
S39	weight N2 (reduc* OR loss OR lose OR lost OR losing OR gain* OR increas* OR chang* OR related)
S38	"over weight"
S37	overweight
S36	obes*
S35	DE "Overweight" OR DE "Obesity" OR DE "Body Mass Index"

S34	S22 OR S23 OR S24 OR S25 OR S26 OR S27 OR S28 OR S29 OR S30 OR S31 OR S32 OR S33
S33	DE "Pediatrics"
S32	"out of school time"
S31	"after school" OR childcare OR "child care"
S30	(elementary OR primary OR grade OR grammar OR pre) N2 school*
S29	youth* OR juvenile*
S28	preschool*
S27	pediatric* OR paediatric*
S26	boy*
S25	girl*
S24	child*
S23	(adolescen* OR pubert* OR pubescen* OR teen*) OR (preadolescen* OR prepubert* OR prepubescen* OR preteen*)
S22	'pre' W1(adolescen* OR pubert* OR pubescen* OR teen* OR school*)
S21	(S1 OR S2 OR S3 OR S4 OR S5 OR S6 OR S7 OR S8 OR S9 OR S10 OR S11 OR S12 OR S13 OR S14 OR S15 OR S16 OR S17 OR S18 OR S19 OR S20)
S20	(behavior* OR behaviour*) N2 chang*
S19	physical N2 (activit* or fitness or training or conditioning or exertion)
S18	calori*
S17	sedentar*
S16	exercis*
S15	snack*
S14	appetite*
S13	meal*
S12	feeding
S11	food*
S10	intake
S9	eating
S8	nutritive
S7	nutrition*
S6	diet*
S5	program*
S4	intervention*
S3	reduc*
S2	prevent*
S1	DE "Prevention" OR DE "Early Intervention"

Epistemonikos.org

(title:((title:(obes* OR overweight OR "overweight" OR adipos* OR weight OR overnutrition OR "over nutrition" OR BMI OR "body mass") OR abstract:(obes* OR overweight OR "overweight" OR adipos* OR weight OR overnutrition OR "over nutrition" OR BMI OR "body mass")) AND (title:(preadolescen* OR prepubert* OR prepubescen* OR preteen* OR preschool* OR pre-adolescen* OR pre-pubert* OR pre-pubescen* OR preteen* OR pre-school* OR adolescen* OR pubert* OR pubescen* OR teen* OR child* OR girl* OR boy* OR pediatric* OR paediatric* OR youth* OR juvenile* OR "elementary school" OR "primary school" OR "grade school" OR "grammar school" OR "after school" OR "out of school time" OR childcare OR "child care" OR

child-care) OR abstract:(preadolescen* OR prepubert* OR prepubescen* OR preteen* OR preschool* OR pre-adolescen* OR pre-pubert* OR pre-pubescen* OR preteen* OR pre-school* OR adolescen* OR pubert* OR pubescen* OR teen* OR child* OR girl* OR boy* OR pediatric* OR paediatric* OR youth* OR juvenile* OR "elementary school" OR "primary school" OR "grade school" OR "grammar school" OR "after school" OR "out of school time" OR childcare OR "child care" OR child-care)) AND (title:(prevent* OR reduc* OR intervention* OR program* OR diet* OR nutrition* OR nutritive OR eating OR intake OR food* OR feeding OR meal* OR appetite OR snack* OR exercise* OR sedentar* OR calori* OR "physical activity" OR "physical activities" OR "physical fitness" OR "physical training" OR "physical conditioning" OR "physical exertion" OR "behavior change" OR "behavior changes" OR "behaviour change" OR "behaviour changes") OR abstract:(prevent* OR reduc* OR intervention* OR program* OR diet* OR nutrition* OR nutritive OR eating OR intake OR food* OR feeding OR meal* OR appetite OR snack* OR exercise* OR sedentar* OR calori* OR "physical activity" OR "physical activities" OR "physical fitness" OR "physical training" OR "physical conditioning" OR "physical exertion" OR "behavior change" OR "behavior changes" OR "behaviour change" OR "behaviour changes")) AND (title:(parent* OR maternal OR paternal OR mother* OR father* OR "family based" OR caregiver* OR "care giver" OR "care givers" OR carer* OR "care provider" OR "care providers" OR guardian*) OR abstract:(parent* OR maternal OR paternal OR mother* OR father* OR "family based" OR caregiver OR "care giver" OR "care givers*" OR carer* OR "care provider" OR "care providers" OR guardian*))) OR abstract:((title:(obes* OR overweight OR "overweight" OR adipos* OR weight OR overnutrition OR "over nutrition" OR BMI OR "body mass") OR abstract:(obes* OR overweight OR "overweight" OR adipos* OR weight OR overnutrition OR "over nutrition" OR BMI OR "body mass")) AND (title:(preadolescen* OR prepubert* OR prepubescen* OR preteen* OR preschool* OR pre-adolescen* OR pre-pubert* OR pre-pubescen* OR preteen* OR pre-school* OR adolescen* OR pubert* OR pubescen* OR teen* OR child* OR girl* OR boy* OR pediatric* OR paediatric* OR youth* OR juvenile* OR "elementary school" OR "primary school" OR "grade school" OR "grammar school" OR "after school" OR "out of school time" OR childcare OR "child care" OR child-care) OR abstract:(preadolescen* OR prepubert* OR prepubescen* OR preteen* OR preschool* OR pre-adolescen* OR pre-pubert* OR pre-pubescen* OR preteen* OR pre-school* OR adolescen* OR pubert* OR pubescen* OR teen* OR child* OR girl* OR boy* OR pediatric* OR paediatric* OR youth* OR juvenile* OR "elementary school" OR "primary school" OR "grade school" OR "grammar school" OR "after school" OR "out of school time" OR childcare OR "child care" OR child-care)) AND (title:(prevent* OR reduc* OR intervention* OR program* OR diet* OR nutrition* OR nutritive OR eating OR intake OR food* OR feeding OR meal* OR appetite OR snack* OR exercise* OR sedentar* OR calori* OR "physical activity" OR "physical activities" OR "physical fitness" OR "physical training" OR "physical conditioning" OR "physical exertion" OR "behavior change" OR "behavior changes" OR "behaviour change" OR "behaviour changes") OR abstract:(prevent* OR reduc* OR intervention* OR program* OR diet* OR nutrition* OR nutritive OR eating OR intake OR food* OR feeding OR meal* OR appetite OR snack* OR exercise* OR sedentar* OR calori* OR "physical activity" OR "physical activities" OR "physical fitness" OR "physical training" OR "physical conditioning" OR "physical exertion" OR "behavior change" OR "behavior changes" OR "behaviour change" OR "behaviour changes")) AND (title:(parent* OR maternal OR paternal OR mother* OR father* OR "family based" OR caregiver* OR "care giver" OR "care givers" OR carer* OR "care provider" OR "care providers" OR guardian*) OR abstract:(parent* OR maternal OR paternal OR mother* OR father* OR "family based" OR caregiver OR "care giver" OR "care givers*" OR carer* OR "care provider" OR "care providers" OR guardian*))))
