# Supplementary material for: Parent Involvement in Diet or Physical Activity Interventions to Treat or Prevent Childhood Obesity: An Umbrella Review
Source: Nutrients. 2021 Sep 16;13(9):3227. doi: 10.3390/nu13093227 (PMC8464903; doi:10.3390/nu13093227)
Supplement: Supplementary file 1 [file nutrients-13-03227-s001.zip › nutrients-1320147-supplementary/Supplementary Files/Table S2. Excluded Studies with Reasons.pdf]

Supplementary Table S2. Articles Excluded after Full Text Review and Reasons for Exclusion

	Author, Publication Year	Reason(s) for Exclusion
1	Agaronov, 2018	BMI not an outcome measure
2	AlMarzooqi, 2011	Not a systematic review
3	Altman, 2015	Not a systematic review
4	Antwi, 2013	Age out of range
5	Ash, 2017	Age out of range
6	Azabedo 2016	No parental component
7	Barnes, 2018	Age out of range
8	Barr-Anderson, 2013	Age out of range
9	Bates 2018	Associations only (no interventions)
10	Berge, 2011	Age out of range
11	Berry, 2004	Age out of range, Not a systematic review
12	Black, 2017	Age out of range, No parental component, Missing outcome criteria
13	Bond, 2009	Age out of range
14	Bond, 2011	Age out of range
15	Bonilla, 2017	Article in Spanish
16	Borrelli, 2015	Age out of range
17	Branscum, 2011	Age out of range
18	Brigden, 2019	BMI not an outcome measure, Missing outcome criteria
19	Brown, 2019	No parental component
20	Brunton, 2003	Age out of range
21	Burchett, 2018	Age out of range
22	Campbell, 2007	Age out of range
23	Collins, 2013	Age out of range
24	Colquitt, 2016	Age out of range
25	D'Onise, 2010	Age out of range
26	Dabas, 2018	Not a systematic review
27	De Niet, 2011	Not a systematic review
28	Dellert, 2014	Age out of range
29	Durbin, 2018	Age out of range
30	Edmunds, 2004	Not a systematic review, Could not be found
31	Ells, 2018	Not a systematic review (umbrella review)
32	Foster, 2015	Age out of range
33	Gao, 2008	Age out of range
34	Gerards, 2011	Age out of range
35	Golley, 2011	Age out of range
36	Guerra, 2016	Not a systematic review
37	Hamel, 2011	Age out of range
38	Hammersley, 2016	Age out of range
39	Hartman, 2011	Age out of range
40	Hesketh, 2010	Age out of range, BMI not an outcome measure
41	Hesketh, 2017	Age out of range
42	Hingle, 2010	Age out of range

43	Ho, 2012	Age out of range
44	Ickes, 2014	Age out of range
45	Inella, 2020	Age out of range
46	Jalali, 2016	Age out of range
47	Janicke, 2014	Age out of range
48	Jull, 2013	Age out of range
49	Kader, 2015	Age out of range
50	Kattemannk, 2011	Not a systematic review
51	Katz, 2008	Age out of range
52	Kelishadi, 2014	Age out of range
53	Kim, 2019	Age out of range
54	Kitzmann, 2011	Not a systematic review
55	Knowlden, 2012	Age out of range
56	Kornet, 2017	Age out of range
57	Kothandan, 2014	Age out of range
58	Kuhl, 2012	BMI not an outcome measure, Not a systematic review
59	Larson, 2011	Age out of range
60	Lee, 2016	No placebo group described
61	Lindsay, 2006	Not a systematic review
62	Ling, 2016	Age out of range, Missing outcome criteria
63	Ling, 2017	Age out of range
64	Lobelo, 2013	Age out of range
65	Luybli, 2019	Not a systematic review
66	Masoumi, 2017	Not a systematic review
67	McCambridge, 2006	BMI not an outcome measure, Not a systematic review
68	Mehdizadeh, 2020	Age out of range
69	Meiklejohn, 2016	Age out of range
70	Melnyk, 2005	Not a systematic review
71	Mikkelsen, 2014	BMI not an outcome measure in all studies
72	Monasta, 2011	Age out of range
73	Morris, 2015	Age out of range
74	Nga, 2019	Not a systematic review
75	Niemeier, 2012	Age out of range
76	Pamungkas, 2019	Age not reported
77	Perez-Morales, 2009	Article in Spanish
78	Raber, 2016	Age out of range
79	Rajjo, 2017	Not a systematic review
80	Reed, 2015	Age out of range, Not a systematic review
81	Rodriquez, 2014	Article in Spanish
82	Russell, 2016	Age out of range
83	Saguil, 2012	Age out of range, Not a systematic review
84	Schlechter, 2016	Age not reported, BMI not an outcome measure
85	Seo, 2010	Age out of range
86	Sharma, 2007	Age out of range
87	Shirley, 2015	Age out of range, Not a systematic review

88	Showell, 2013	Age out of range
89	Silveira, 2011	Age out of range
90	Skouteris, 2011a	Age out of range
91	Skouteris, 2011b	Conference Abstract
92	Skouteris, 2012	Age out of range
93	Small, 2013	Missing outcome criteria, No placebo group described
94	Snuggs, 2019	Age out of range
95	Spinola e Castro, 2014	Not a systematic review
96	Sreevatsava, 2013	Age out of range
97	Stacey, 2017	Not a systematic review
98	Steyn, 2009	Age out of range
99	Stice, 2006	Age out of range
100	Sung-Chan, 2013	Age out of range
101	Swanson, 2011	Age out of range
102	Thury,2015	Not a systematic review
103	Upton, 2014	Age out of range
104	Van de Kolk, 2019	Age out of range
105	Van der Kruk, 2013	Age out of range
106	Van Lippevelde, 2012	Age out of range
107	Vasques, 2014	Age out of range
108	Ventura, 2008	Age not reported, Not a systematic review
109	Ward, 2017	Age out of range
110	Waters, 2011	Age out of range
111	Wilson, 2014	Not a systematic review
112	Wofford, 2008	Age out of range, Not a systematic review
113	Yavuz, 2015	Age out of range, Not a systematic review
114	Young, 2018	Age out of range, Missing outcome criteria
115	Zenzen, 2009	Age out of range, Not a systematic review
