# Supplementary material for: Parent Involvement in Diet or Physical Activity Interventions to Treat or Prevent Childhood Obesity: An Umbrella Review
Source: Nutrients. 2021 Sep 16;13(9):3227. doi: 10.3390/nu13093227 (PMC8464903; doi:10.3390/nu13093227)
Supplement: Supplementary file 1 [file nutrients-13-03227-s001.zip › nutrients-1320147-supplementary/Supplementary Files/Table S3. Primary Articles Included.pdf]

Supplementary Table S3. Primary Articles Included for each Systematic Review and/or Meta-Analysis

	**Number of times cited**	**Ewald**	**Jang**	**Loveman**	**McLean**	**Mead**	**Oude Luttikhuis**	**Sbruzzi**	**Young**	**Gori**	**Laws**	**Nixon**	**Oosterhoff**	**Sobol-Goldberg**	**Verjans-Janssen**
Adams 2008	1											**X**			
Ahamed 2007	1												**X**		
Alexander 2014	1														**X**
Alexandrov 1992	1												**X**		
Amaro 2006	1												**X**		
Angelopoulos 2009	2												**X**		**X**
Annesi 2013	1												**X**		
Aragona 1975	1			**X**											
Arauz Boudreau 2013	1					**X**									
Armeno 2011	1									**X**					
Bacardi-Gascon 2012	1														**X**
Baranowski 2003	1									**X**					
Barbeau 2007	1												**X**		
Bayer 2009	1											**X**			
Beech 2003	2								**X**	**X**					
Bocca 2013	1									**X**					
Boutelle 2011	2	**X**		**X**											
Boutelle 2014	1					**X**									
Boutelle NCT01197443	1	**X**													
Brandstetter 2012	1												**X**		
Bryant 2011	1					**X**									
Burke 1998	1												**X**		
Bush 1989	1												**X**		
Caballero 2003	1												**X**		
Cao 2015	1														**X**
Carrel 2005	1												**X**		
Centis 2012	1														**X**
Chan 2012	1												**X**		
Chomitz 2010	1														**X**
Collins 2011	2	**X**		**X**											
Coppins 2011	1					**X**									
Crespo 2012	2												**X**		**X**
Croker 2011	2							**X**		**X**					

Croker 2012	1					X									
Danielzik 2007	1											X			
Davis 2013	1					X									
Davoli 2013	1					X									
de Coen 2012	1												X		
de Heer 2011	1												X		
de Ruyter 2012	1									X					
Dennison 2004	1												X		
Diaz 2010	1					X									
Donnelly 2009	1												X		
Duncan 2009	1												X		
Dzewaltowski 2010	1												X		
Eather 2013	1												X		
Eddy Ives 2012	1					X									
Elliakim 2007	1												X		
Endevelt 2014	1									X					
Epstein 1981	1				X										
Epstein 1982	1								X						
Epstein 1985a	1								X						
Epstein 1985b	1								X						
Epstein 1986	1				X										
Epstein 1987a	1				X										
Epstein 1987b	1				X										
Epstein 1987c	1				X										
Epstein 1990	1				X										
Epstein 1994	1				X										
Epstein 2000a	2					X			X						
Epstein 2000b	1								X						
Epstein 2004	1								X						
Epstein 2005	1						X								
Esfarjani 2013	1			X											
Estabrooks 2009	3		X	X				X							
Fairclough 2013	1												X		
Feng 2016	1														X
Fitzgibbon 2005	1										X				
Fitzgibbon 2006	3										X	X	X		
Fitzgibbon 2011	2										X		X		
Fitzgibbon 2013	1										X				
Flodmark 1993	2				X		X								
Foster 2008	1												X		

Foster 2010	1											X			
Gentile 2009	2									X			X		
Gillis 2007	1					X									
Golan 1998a	1	X													
Golan 1998b	1	X													
Golan 2004	1	X													
Golan 2006	3	X		X			X								
Golley 2007	3			X			X	X							
Graf 2006	1												X		
Graf 2008	1												X		
Graham 2008	1											X			
Graves 1988	1								X						
Grydeland 2014	1												X		
Gunnarsdottir 2011	1					X									
Haire-Joshu 2010	1									X					
Hansen 1991	1												X		
Harrell 1999	1												X		
Harrison 2006	1												X		
Henaghan 2008	1												X		
Hills 1988	1								X						
Ho 2016	1					X									
Hoffman 2011	1												X		
Hopper 2005	1												X		
Hu 2010	1											X			
Hughes 2008	1						X								
Israel 1984	1								X						
Israel 1985	2				X				X						
Israel 1986	1								X						
Israel 1990	1								X						
Israel 1994	1				X										
James 2004	1												X		
Janicke 2008	2	X		X											
Janicke 2009	1	X													
Janicke 2011	1	X													
Jansen 2011	3		X	X									X		
Jiang 2007	2												X		X
Johnston 2013	1												X		
Jouret 2009	2										X		X		
Kain 2004	1														X
Kalarchian 2009	2					X		X							

Kalavainen 2007	3					**X**	**X**	**X**							
Kipping 2008	1												**X**		
Kirk 2012	1					**X**									
Kirschenbaum 1984	2				**X**				**X**						
Kriemler 2010	2											**X**	**X**		
Lazaar 2007	1												**X**		
Levine 2001	1								**X**						
Li 2010	1												**X**		
Li 2014	1														**X**
Lison 2012	1					**X**									
Llargues 2012	1												**X**		
Lloyd 2012	1												**X**		
Lohman 2003	1												**X**		
Looney 2014	1					**X**									
Maddison 2014	1					**X**									
Magarey 2011	2		**X**	**X**											
Magnusson 2012	1												**X**		
Manios 1999	2												**X**		**X**
Manios 2002	2											**X**	**X**		
Marcus 2009	1												**X**		
Markert 2014	1					**X**									
Martinez-Vizcaino 2008	1												**X**		
Mazzeo 2012	1		**X**												
Mazzeo 2014	2		**X**	**X**											
McCallum 2007	2					**X**	**X**								
McMurray 2002	1												**X**		
Meng 2013	1												**X**		
Mirza 2013	1					**X**									
Mo-Suwan 1998	2											**X**	**X**		
Moens 2012	2		**X**	**X**											
Muckelbauer 2009	1												**X**		
Munsch 2008	3	**X**		**X**			**X**								
Nader 1999	1												**X**		
Nemet 2005	1					**X**									
Nemet 2011	1												**X**		
Niederer 2013	2										**X**		**X**		
O'Connor 2013	1					**X**									
Okley 2010	1	**X**													
Paineau 2008	1									**X**					
Plachta-Danielzik 2007	1												**X**		

Plachta-Danielzik 2011	1											X			
Rausch Hersovici 2013	1											X			
Raynor 2012a	1			X											
Raynor 2012b	1			X											
Reed 2008	1											X			
Reilly 2006	2										X	X			
Reinehr 2010	1					X									
Resnick 2009	1			X											
Resnicow 2015	1			X											
Robinson 1999	1											X			
Robinson 2003	1									X					
Rodearmel 2007	1					X									
Rosario 2013	1											X			
Sacchetti 2013	1											X			
Sacher 2010	3					X		X		X					
Saelens 2013	1					X									
Sahota 2001	1											X			
Salcedo Aguilar 2010	1											X			
Sallis 1993	1											X			
Salmon 2008	1											X			
Sanigorski 2008	2									X					X
Senediak 1985	1								X						
Serra-Paya 2015	1					X									
Shelton 2007	1		X												
Sichieri 2009	1											X			
Siegrist 2013	2											X			X
Simon 2008	1											X			
Siwik 2013	1					X									
Small 2013	1			X											
Spiegel 2006	1											X			
Stock 2007	2										X	X			
Story 2003	2									X		X			
Story 2012	1											X			
Tarro 2014	1									X					
Taylor 2007	1											X			
Taylor 2015	1					X									
Thivel 2011	1											X			
Toruner 2010	1											X			
Treu 2015	1														X
Trost 2014	1									X					

Van Grieken 2013	1			**X**											
VanDongen 1995	1												**X**		
Wafa 2011	2					**X**		**X**							
Wake 2009	1					**X**									
Wake 2013	1					**X**									
Waling 2010	1							**X**							
Walling 2012	1					**X**									
Walter 1985	1												**X**		
Walther 2009	1												**X**		
Warren 2003	1											**X**			
Warschburger 2016	1					**X**									
Waters 2018	1														**X**
Weigel 2008	1					**X**									
Weintraub 2008	1					**X**									
West 2010	2		**X**	**X**											
Willi 2012	1												**X**		
Williamson 2012	2									**X**			**X**		
Winter 2013	1										**X**				
Wright 2013	1									**X**					
Xu 2015	1														**X**
Xu 2017	1														**X**
Yin 2005	1												**X**		
Yin 2012	1												**X**		
	**1.23**	**12**	**8**	**20**	**11**	**38**	**8**	**8**	**16**	**18**	**7**	**12**	**89**	**8***	**18**

*Eight studies are described/summarized in the publication, but the identity of the eight studies could not be ascertained.
